# Therapeutic Potential and Effective Components of the Chinese Herb Gardeniae Fructus in the Treatment of Senile Disease

**DOI:** 10.14336/AD.2018.0112

**Published:** 2018-12-04

**Authors:** Shichao Lv, Yang Ding, Haiping Zhao, Shihao Liu, Junping Zhang, Jun Wang

**Affiliations:** ^1^Institute of Basic Theory, China Academy of Chinese Medical Sciences, Beijing, China; ^2^Department of Geriatric Medicine, First Teaching Hospital of Tianjin University of Traditional Chinese Medicine, Tianjin, China; ^3^Digestive Disease Center, Beijing Hospital of Traditional Chinese Medicine Affiliated to Capital Medical University, Beijing, China; ^4^Cerebrovascular Diseases Research Institute, Xuanwu Hospital of Capital Medical University, Beijing, China; ^5^Department of Cell and Developmental Biology, School of Molecular and Cellular Biology, University of Illinois at Urbana-Champaign, USA

**Keywords:** age-related diseases, gardenia, geniposide, mechanism, Senile Disease, Chinese Herb

## Abstract

Gardeniae fructus (GF), an evergreen Rubiaceae shrub, is one of the most commonly used Chinese herbs in traditional Chinese medicine (TCM) and has been used for over a thousand years. It is usually prescribed for the treatment of brain aging, vascular aging, bone and joint aging, and other age-related diseases. It has been demonstrated that several effective compounds of GF, such as geniposide, genipin and crocin, have neuroprotective or related activities which are involved in senile disease treatment. These bioactivities include the mitochondrion dysfunction, antioxidative activity, apoptosis regulation and an anti-inflammatory activity, which related to multiple signaling pathways such as the nuclear factor-κB pathway, AMP-activated protein kinase signaling pathway, and the mitogen-activated protein kinase pathway. To lay the ground for fully elucidating the potential mechanisms of GF in treating age-related pathologies, we summarized the available research conducted in the last fifteen years about GF and its effective components, which have been studied *in vivo *and *in vitro*

The lifespan of human beings has been prolonged with the modernization and development of medical progress. In the last century, developed countries have benefited from medical advances, improvements in public healthcare systems and better living conditions derived from their socioeconomic power, which have helped achieve a marked increase in life expectancy [1]. However, age-related diseases are still a big challenge for us. Aging is a long, gradual process of functional decline which may not necessarily result in diseases that need to be treated [2]. The number of people suffering from age-related diseases is anticipated to almost double over the next two decades [3]. According to the China Aging Development Report (2013), the severity of the aging population in China is rarely seen elsewhere in the world. By 2030, China’s aging population (aged 60 and above) is expected to reach 400 million, which is almost equivalent to the total population of 15 EU countries [4].

According to the systemic theory, aging is associated with a decline in the function of essential organ systems [5]. Many physiological and pathological mechanisms are involved in the aging process. The free radical theory of aging postulates that the production of intracellular reactive oxygen species (ROS) is the major determinant of lifespan [6]. Mitochondrial dysfunction is the central in the aging theories, as age-related changes in mitochondria are likely to impair a host of cellular physiological functions in parallel and contribute to the development of all common age-related diseases [7]. A defect in mitochondrial respiratory enzymes can increase mitochondrial production of ROS, causing further mitochondrial damage, leading to a further decline in cellular and organ function that can eventually progress to death [8]. Several important pro-inflammatory mediators, such as nuclear factor-κB (NF-κB), inducible nitric oxide synthase and cyclooxygenase, are known to increase ROS production, which leads to DNA and tissue damage and thus results in age-related disease [9]. Low-grade inflammation is also a hallmark of aging, and the systemic level of inflammation is negatively correlated with human longevity [10]. Recent investigations revealed a link between autophagy and aging in yeast, most notably because autophagy genes are required for TOR inhibition to extend chronological lifespan [11, 12], and because spermidine, a pharmacological activator of autophagy, increases lifespan via an autophagy-dependent mechanism [13].

Many Chinese herbs have antiaging properties and could intervene aging-associated disorders such as Ginseng, Huangqi and Herba Cistanches, et al [14-16]. The Chinese herb Gardeniae fructus (GF) is an evergreen Rubiaceae shrub, which is widely used in Asian countries as a complementary and alternative therapy. As a kind of traditional Chinese medicine with the effect of clearing heat and detoxifying, GF has been used in traditional Chinese medicine (TCM) due to the homeostatic, antiphlogistic, analgesic and antipyretic effects [17]. Like other Chinese herbs with clearing heat and detoxifying effects such as Rhizoma Coptidis and Baicalin [18-19], GF also has antiaging effect. The main components of GF include geniposide and crocin [20] which exhibit antioxidant, anti-inflammatory and hypolipidemic activities [21-23]. Several important pathologies and pathways related to aging, such as oxidative stress, mitochondrial malfunction, and mTOR signaling pathways were confirmed to be regulated by GF or its components, particularly geniposide [24-26]. Thus, we regard GF as a promising candidate for the prevention and treatment of aging and age-associated disorders by targeting multiple age-associated signaling pathways. In this review, we aim to summarize the possible mechanisms and pathways related to the anti-aging activity of GF and its components in combating age-related diseases.

**Figure 1.**
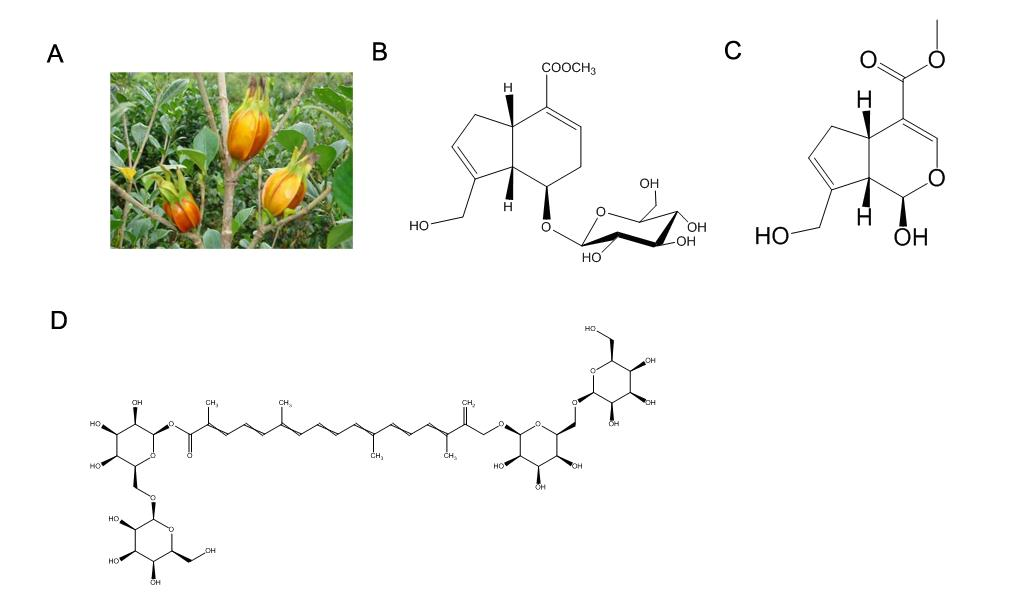
Chemical structures of the main bioactive components of Gardeniae fructus (GF). (A) Photo of Gardenia. (B) Geniposide (C_17_H_24_O_10_, molecular weight: 300); (C) genipin (C_11_H_14_O_5_, molecular weight: 226.23); (D) crocin (C_44_H_64_O_24_, molecular weight: 976.96).

## 1. Modern pharmacological research on the main constituents of gardenia

### 0.1A general overview of gardenia and its bioactive components.

Gardeniae fructus (Fig. 1A; Chinese herbal name: Zhizi), is firstly recorded in the book named ‘Shen Nong’s Herbal Classic’, and is mainly distributed in tropical zones and subtropical regions of China. Flora of China pointed out that there were seven types of Gardenia plants in China: Zhizi (*G. J. E.*), Hainan Zhizi (*G. Hananensis* Merr.), Xiaye Zhizi (*G. stenophylla* Merr.), Shiye Zhizi (*G. angkorensis* Pitard), and Dahuang Zhizi (*G. sootepensis* Hutchins.) with two varieties (Baichan Zhizi, or Chongban Zhizi, *G. jasminoides* Ellis var. fortuniana (Lindl.) Hara and *G. jasminoides* var. jasminoides) [27]. Among the different species of the Gardenia genus, the mainstream species is Zhizi (*G. J. E.*) which is usually used for medicine in China [28]. Some scholars used crocin, an effective component of Gardenia, to observe the differences in the effective components among these species. The results showed that the species was not the only factor that affected the content of the effective components; the storage conditions also had an influence. Prolonged storage time, light or high temperature could decrease the contents of effective components [28]. A number of new iridoid glycosides have been identified, which are major bioactive constituents, such as geniposide, genipin, geniposidic acid, crocetin and genipin-1-β-D-gentiobioside [29]. Geniposide (Fig. 1B) is a major iridoid compound present in *G. jasminoides* fruits, and genipin, an aglycone of geniposide, is the only iridoid aglycone commercially available in relatively large amounts. Geniposide is hydrolyzed to the aglycone genipin by h-D-glucosidases in the intestine and the liver [30]. Genipin (Fig. 1C) has a molecular weight of 226 and a white crystalline structure. It is soluble in ethanol and ethyl acetate and slightly soluble in water [31]. Genipin generates both color and fluorescence in a single reaction with biopolymers containing primary amine groups. The reaction between genipin and collagen induces the formation of cyclic structures, which serve as the intramolecular and intermolecular crosslinks between collagen molecules within fibers [32]. Crocin (Fig. 1D), a 8,8'diapo-8,8'-carotenic acid, belongs to the carotenoid family. It is characterized by a diterpenic and symmetrical structure with alternating double bonds and four methyl groups, and is slightly soluble in water [33]. Crocin is a part of the group of hydrophilic carotenoids with either mono- or di-glycosyl polyene esters of crocetin with D-glucose and/or D-gentiobiose as carbohydrate residues [34]. A variety of traditional Chinese herbal preparations contain GF, such as Longdan Xiegan Pill [35], Yinchenhao Decoction [36], or Zhizichi Decoction [37]. In the traditional Chinese patent medicine, GF has been used as an ingredient in Huanglian Jiedu Decoction [38], Tongluo Jiunao Injection [39], and Xingnaojing Injection [40].

### 1.2 Pharmacokinetic profile

The pharmacokinetic profile of GF bioactive components such as geniposide or genipin has been well studied in humans and animals. Geniposide is a water-soluble iridoid glycoside component found in GF, but geniposide itself is not regarded as a major active ingredient of GF extracts [41]. Geniposide has been shown to be hydrolyzed by β-D-glucosidases into genipin in the intestine. Genipin is liposoluble, and this property enables genipin to easily permeate into intestinal mucosa and facilitates absorption. It was found that intestinal bacteria in animals could transform geniposide to its aglycone genipin [30]. Some studies have shown that after oral administration of genipin or GF contained decoction, genipin sulfate is a major metabolite in the bloodstream, whereas the precursor forms of genipin and geniposide were not detected [42]. When used in combination with other herbs, the absorption of geniposide could change. Berberine may not affect the absorption of geniposide, whereas baicalin increase the absorption of geniposide. In addition, the function of berberine is to inhibit the positive effect of baicalin on geniposide uptake in the body [43]. The absolute bioavailability of geniposide was 76.14% following intranasal administration combined with natural borneol. Compared with the intranasal administration of geniposide alone, geniposide can be absorbed rapidly in the nasal cavity combined with natural borneol [44]. In various physiological or pathological animal models, the effects of geniposide absorption also varied greatly. Specifically, the effects of geniposide absorption in middle cerebral artery occlusion injured rats were better than in normal rats [43] (Table 1).

## 2. The anti-aging effect of Gardenia and its components

### 2.1 Brain aging

#### Alzheimer's disease

Alzheimer's disease (AD), characterized by memory deficits and cognitive decline, is a progressive neurodegenerative disorder. In an AD mouse model, geniposide ameliorated cognitive defects in middle-aged mice [45]. Lv et al. reported that treatment with geniposide suppressed RAGE-dependent signaling, the production of interleukin (IL)-1β and tumor necrosis factor-α and cerebral Aβ accumulation in an AD mouse model. Geniposide improved learning and memory in model mice [46]. Geniposide trafficked in cultured hippocampal neurons and improved mitochondrial motility, alleviated Aβ-induced axonal mitochondrial abnormalities by increasing axonal mitochondrial density and length. In neurons and APPswe/PS1dE9 mice, decreases in synapse-related proteins were ameliorated by geniposide [47]. A study in APP/PS transgenic AD mice showed that the leptin antagonist influenced the expression of secretases and the activities of leptin signaling molecules associated with the production of Aβ 1-42. Geniposide may regulate the production of Aβ 1-42 via leptin signaling [48]. In AD transgenic mouse model, geniposide at the concentrations of 2.5, 5 and 10 μM protected cultured primary cortical neurons from Aβ-mediated mitochondrial dysfunction in a dose-dependent manner by recovering mitochondrial membrane potential, promoting ATP generation and increasing cytochrome c oxidase and caspase 3/9 activity by reducing cytochrome c leakage and ROS production, and by inhibiting apoptosis [49]. In an insulin-deficient APP/PS1 transgenic mouse model, geniposide enhanced the role of insulin on the phosphorylation of GSK-3b, Akt and tau in primary cultured cortical neurons [50].

**Table 1:** The anti-aging effects of gardenia and its components.

Model	Tissue	Mechanisms	Bioactive component	Ref.
Mice	Brain	MAPK signaling pathway↓ ChAT↑, AChE↓	Geniposide	[62]
Mice	Brain	RAGE-dependent signaling, TNF-α, IL-1β and cerebral Aβ accumulation↓	Geniposide	[46]
APPswe/PS1dE9 mice	Brain	Increase axonal mitochondrial density and length	Geniposide	[47]
APP/PS transgenic AD mice	Brain	Induce the phosphorylation of JAK2 and STAT3	Geniposide	[48]
Insulin-deficient APP/PS1 transgenic mouse	Brain	The phosphorylation of GSK-3β↑ The phosphorylated level of tau↓	Geniposide	[49]
The MPTP mouse model of PD	Brain	Bcl-2↓ Bax↑	Geniposide	[51]
PD mouse	Brain	Block microRNA-21/lysosome-associated membrane protein 2A interaction	Geniposide	[52]
Spontaneously Hypertensive rats	kidney	blood pressure, serum creatinine, blood urea nitrogen, cell proliferation, ROS generation↓	Genipin	[56]
C57/B6 mice	Heart	AMPKα↑ mammalian target of rapamycin, ERK and endoplasmic reticulum stress↓	Geniposide	[57]
Rabbit	Artery	ECs shedding, the plaque area, intima/media thickness ratio, intimal foam cells number↓	Geniposidic acid	[58]
Mouse and rat	Artery	length of tail thrombus, platelet aggregation, venous thrombosis↓	GJ-ext?Geniposide and genipin	[60[
Aging rat	Liver	cellular ROS overproduction, MMP, ATP, Akt phosphorylation↓ glucose consumption, glycogen synthesis↑	Genipin	[73]
Collagen-induced arthritis rats	Joint	IL-4, transforming growth factor-beta 1↑ IL-6, IL-17, P-Raf, P-MEK, P-Erk1/2↓	Geniposide	[68]
Rat	Ankle joint	Swelling ratio↓	Geniposide	[72]

Note: Treg, regulatory T; p-JNK, phospho-JNK; IL-6, interleukin 6; AchE, acetylcholin esterase; TNF, tumor necrosis factor;MPTP, 1-Methyl-4-phenyl-1,2,3,6-tetrahydropyridine; LDL-c: low-density lipoprotein cholesterol; AMPKα, 5′-AMP-activated protein kinase-α; ECs, endothelial cells; GJ-ext, extract of G. jasminoides; SOD, superoxide dismutase; NOS, nitric oxide synthase; fgf, Fibroblast growth factor; PXN, paxillin; ROS, reactive oxygen species; MMP, mitochondrial membrane potential; ATP, adenosine tri-phosphate.

#### Parkinson's disease

The neuroprotective effects of geniposide were also studied in the MPTP mouse model of Parkinson's disease (PD). After MPTP treatment, geniposide was administered (100 mg/kg intraperitoneal) for 8 days. Geniposide treatment reduced the apoptosis signaling molecule Bcl-2 and increased the levels of growth factor signaling molecule Bax [51]. Geniposide also reduced α-synuclein by blocking the microRNA-21/lysosome-associated membrane protein 2A interaction in PD models [52].

### 2.2 Vascular aging effect

#### Cardiovascular aging

Gardenia and its components, especially geniposide, possess unique pharmacological activities in cardiovascular disorders. Initial studies about the atherosclerosis treatment with GF showed that gardenia inhibited the development of atherosclerosis in ApoE-knockout mice. Geniposide inhibited dickkopf-related protein-1 and increased Wnt1 expression, while upregulating the expression of foxp3, decreasing the numbers of dendritic cells (DC), and inhibiting DC maturation and infiltration into lesions in bone marrow [53-55]. In hypoxia/reoxygenation injured H9c2 cells, pretreatment with geniposide increased cell viability, decreased lactate dehydrogenase levels in the supernatant, and inhibited cardiomyocyte apoptosis caused by hypoxia/reperfusion. Furthermore, geniposide reversed mitochondrial dysfunction by decreasing oxidative stress products (reactive oxygen species/reactive mitrogen species and malondialdehyde) by increasing antioxidative enzyme level, improving mitochondrial morphology, attenuating mitochondrial calcium overload and blunting depolarization of the mitochondrial membrane [25]. In addition, geniposide at a range of 10-80 µM improved H9c2 cell viability, with 40 µM being the optimal dosage. Another study of spontaneously hypertensive rats suggested that genipin not only decreased blood pressure, but also improved renal function, as shown by decreased blood urea nitrogen and serum creatinine, as well as urinary microalbumin and N-acetyl-β-D-glucosaminidase [56]. In C57/B6 mice with transverse aorta constriction, geniposide inhibited the hypertrophic response induced by constriction of the transverse aorta or by isoprenaline. Activation of 5'-AMP-activated protein kinase-α (AMPKα) and inhibition of mammalian target of rapamycin, extracellular regulated protein kinases and endoplasmic reticulum stress were observed in hypertrophic hearts treated with geniposide [57]. In the atherosclerosis rabbit model, geniposidic acid improved the plaque area, intima/media thickness ratio, and intimal foam cell number [58]. Genipin inhibited TNF-α-induced vascular smooth muscle cells proliferation and migration in a dose-dependent manner. Genipin prevented Akt phosphorylation and ERK/ mitogen activated protein kinase (MAPK), while c - Jun N - terminal kinase and p38 MAPK were unchanged [59]. The antithrombotic activities of the aqueous extracted geniposide were studied in mouse and rat models. Geniposide decreased the length of tail thrombus, improved thrombosis and inhibited platelet aggregation induced by thrombin/collagen [60]. In lipopolysaccharide (LPS)-induced human umbilical vein endothelial cells (HUVECs), geniposide inhibited LPS-induced expression of IL-8 and IL-6 at the translation and transcription levels. Additionally, geniposide suppressed the adhesion of U937 monocyte to HUVECs as well as LPS-induced HUVEC migration [61].

#### Cerebrovascular aging

In a study of chronic cerebral ischemia, geniposide protected the brain from injury and improved learning and memory [62]. In addition, in the *in vitro* model of cerebral ischemia induced by oxygen-glucose-deprivation (OGD) in brain microvascular endothelial cells (BMEC), the results showed that geniposide decreased the production of monocyte chemotactic protein 1, IL-8 and IL-1β, and downregulated the expression of P2Y 14 receptor and the downstream ERK1/2 signaling pathway [63]. Geniposide displayed a neuroprotective effect on ischemia/ reperfusion-injured rats *in vivo *and inhibited OGD-induced activation of microglial cells by attenuating inflammatory factors and nuclear factor-κB (NF-κB) activation *in vitro* [64]. In the mouse model of brain damage induced by focal cerebral ischemia/reperfusion, jasminoidin and ursodeoxycholic acid were used to treat focal cerebral ischemia and reperfusion injury, which was characterized by the expression of the gene *Hspa1a*, genes *Fgf12*, *Rara*, and *Map3k4* were up-regulated and gene *PXN *was down-regulated, and the p53 pathway was activated [65]. In deprivation-exposed hippocampal slices cultured with damaged neuronal cells, neuronal cell death of both the granular cell layer (dendate gyrus region) and the pyramidal (CA 1 plus 3 region) region was ameliorated by 10, 50 and 100 µM geniposide in a dose-dependent manner [66] (Table 2).

### 2.3 Bone and joint aging

Chronic arthritis and osteoporosis are becoming serious issues in our aging society. GF has been used in the treatment of arthritis and related diseases. Geniposide could reduce the production of inflammatory cytokines and regulate immunity, which played critical roles in the treatment of arthritis [67, 68]. In TCM, GF can be made into an extract to treat arthritis by external application to decrease the content of IL-1β and TNF-α. Gardenia in combination with other Chinese herbs based on TCM theory could also have the therapeutic effect on arthritis [69]. In addition, geniposide was a useful drug for the treatment of postmenopausal osteoporosis as it inhibited c-Fos protein proteolysis and NF-κB activation [70]. Geniposide might also ameliorate ligament injuries and reduce the risk of degenerative joint disease [71, 72]. In addition, 25, 50, 100 and 200 µM geniposide dose-dependently improved cell survival.

**Table 2:** The function and mechanisms of the neuroprotective effects of geniposide.

Disease	Cells/tissues	Effects	Mechanisms	Ref.
AD	Mice	Enhance cholinergic neurotransmission	MAPK signaling pathway↓ ChAT↑, AChE↓	[45]
	Mice	Anti-inflammation	RAGE-dependent signaling, TNF-α, IL-1β and cerebral Aβ accumulation↓	[46]
	APPswe/PS1dE9 mice	Improve mitochondrial motility	Increase axonal mitochondrial density and length	[47]
	APP/PS transgenic AD mice	Regulate leptin signaling	Induce the phosphorylation of JAK2 and STAT3	[48]
	AD transgenic mouse	Anti-apoptotic Anti-oxidant	Bcl-2↑ Cytochrome c, caspase-9, caspase-3, Bax and ROS↓	[49]
	Insulin-deficient APP/PS1 transgenic mouse	Enhance insulin signaling	The phosphorylation of GSK-3β↑ The phosphorylated level of tau↓	[50]
PD	The MPTP mouse model of PD	Anti-apoptotic	Bcl-2↓ Bax↑	[51]
	PD mouse	Reduce α-synuclein	Block microRNA-21/lysosome-associated membrane protein 2A interaction	[52]
Cerebrovascular aging	BMECs	Anti-inflammation	The production of MCP-1, IL-8 and IL-1β↓ Expression of P2Y_14_ receptor and ERK1/2 signaling pathway↓	[53]
	Microglial cells	Anti-inflammation	Release of TNF-α, IL-1β, IL-6, IL-8 and IL-10↓ NF-κB activation↓	[64]
	Hippocampal slice	Neuroprotective effect	Ameliorate the neuronal cell death of both the granular and pyramidal cell layer	[66]

### 2.4 Anti-aging effect in aging animals

In natural-aging rats, hepatic tissues show steatosis and reduced glycogen content. Hepatic malondialdehyde level and mitochondrial ROS are higher, and mitochondrial membrane potential (MMP) and ATP level are lower compared with normal control rats. Administration of genipin ameliorated systemic and hepatic insulin resistance, hyperglyceridemia, hepatic steatosis, and alleviated hyperinsulinemia, and relieved hepatic oxidative stress and mitochondrial dysfunction in aging rats [73]. There is mounting evidence to show that changes occurring in the articular cartilage during the development of osteoarthritis are the result of an age-related loss of normal homeostasis. The aging of chondrocytes appears to contribute to the loss of homeostasis [74]. Genipin preserved chondrocyte viability, which suggested that genipin had an anti-aging effect on chondrocytes [75]. In old rat hearts, treatment with genipin at the dose of 5-10 mol/L for 15 min before prolonged ischemia exerted powerful antiradical and antilipoperoxidative activity [76].

## 3. Mechanisms and key components of Gardenia’s anti-aging effect

### 3.1 Mitochondrion mechanism

Damaged mitochondria not only produce less ATP but also release greater numbers of ROS and have a higher propensity to induce apoptosis, and these phenomena are related to cardiac aging [77]. In Aβ-treated neurons and an AD mouse model, geniposide alleviated Aβ-induced axonal mitochondrial abnormalities by increasing axonal mitochondrial density and length and improving mitochondrial motility and trafficking in cultured hippocampal neurons [47]. A study showed that geniposide reversed mitochondrial dysfunction by decreasing oxidative stress products, improving mitochondrial morphology, increasing anti-oxidative enzyme level, blunting depolarization of the mitochondrial membrane and attenuating mitochondrial calcium overload [25]. In the brains of AD patients, the accumulation of Aβ is thought to be related to neuronal mitochondrial dysfunction. A study found that in AD transgenic mouse models, a pharmacologically active compound purified from gardenia, could reverse mitochondrial dysfunction and oxidative stress [49]. When neurons were cultured in ischemia-injured BMECs conditioned media, geniposide decreased cytochrome c release and increased MMP, which suggested a recovery of mitochondrial function [78].

### 3.2 Anti-oxidant activity

There was evidence that SOD^+/-^ mice showed a clear increase in ROS load, but had a normal lifespan [79]. Reactive oxygen species accumulate over time and are the main contributor to the aging process [6]. Gardeniae fructus extract-capped gold nanoparticles effectively attenuated the increase in lysosome content and ROS production, and protected ARPE19 cells from hydrogen peroxide-induced premature senescence [80]. In ischemic brain injury, geniposide exhibited neuroprotective activity by preventing oxygen free radicals, improving the content of SOD, inhibiting nitric oxide synthase and anticholinesterase (AChE) activity and protecting neurons in the hippocampus CA1 and brain cortex [62]. When PC12 cells were exposed to hydrogen peroxide, geniposide enhanced the phosphorylation of Akt308, Akt473, GSK-3beta and PDK1 under conditions of oxidative stress [81]. In cancer treatment, the topical application of geniposide inhibited 12-o-tetradecanoylphorbol-13-acetate (TPA)-induced edema in mice. Pretreatment of mouse skin with various amounts of geniposide caused inhibition of hydrogen peroxide and myeloperoxidase formation by TPA [78].

### 3.3 Anti-inflammatory activity

#### NF-κB signaling 

Inflammation is the defensive response of living tissue in the vascular system to injury. Many studies have shown that Gardenia has anti-inflammatory activity in various diseases. Research on the treatment of arthritic diseases showed that geniposide inhibited colonic inflammation by decreasing the production of proinflammatory mediators, such as TNF-α, IL-1 and IL-6, increasing the level of anti-inflammatory cytokine IL-10 and inhibiting the phosphorylation of p38MAPK-related proteins. Notably, one study suggested that geniposide attenuated DKK1 expression and enhanced Wnt1 signaling in cardiovascular disease, which may be associated with further inhibition of downstream cytokine expression and the inhibition of transcription factor NF-κB [55]. Geniposide could suppress the phosphorylation of inhibitory kappa B (IκBα), NF-κB, p38, ERK and JNK to downregulate the production of TNF-α, IL-1β and IL-6 in LPS-induced mastitis in mice [83].

#### MAPK signaling

The MAPK signaling pathway is involved in many inflammatory diseases. GF reduced IL-6, IL-12, TNF-α and interferon-γ levels in mice with gastric injury, and mediated the p38MAPK signaling pathway in the regulation of damage and repair of the gastric mucosa epithelium [84]. In OGD-induced BMECs, geniposide inhibited the downstream ERK1/2 signaling pathways, and increased the release of proinflammatory cytokines IL-8, MCP-1 and IL-1β, indicating that geniposide attenuated the inflammatory reaction through regulation of the MAPK signaling pathway. The MAPK signaling pathway is also essential in arthritic diseases, as shown by the inhibitions of proinflammatory cytokine IL-6 and IL-17 and decreased expression of p-Raf, p-MEK, and p-Erk1/2 levels by geniposide [68]. In genomics research, MAPK signaling pathway-related genes, such as *Fgf12, Hspa1a, Rara, Map3k4* were found to be involved in the treatment effect of geniposide [65]. During the pathological process of atherosclerosis, genipin inhibited TNF-α-induced VSMC migration and proliferation in a dose-dependent manner by preventing ERK/MAPK and Akt phosphorylation, while JNK and p38 MAPK were unchanged [59]. In addition, geniposide effectively inhibited LPS-induced expression of IL-6 and IL-8 in HUVECs by blocking the activation of NF-κB, degradation of IκBα, and phosphorylation of p38 MAPK and ERK1/2 in HUVECs challenged with LPS [61]. When microglial N9 cells were pre-treated with vehicle or geniposide and exposed to LPS, geniposide blocked the phosphorylation of p38 and ERK1/2, and inhibited the decrease in IκBα [85].

#### AMPK signaling

In digestive system diseases, AMPK activity has a role in epithelial barrier function. Geniposide attenuated LPS-induced epithelial barrier dysfunction by reducing proin?ammatory cytokine release and activating the AMPK signaling pathway. Both AMPK siRNA transfection and AMPK overexpression abrogated geniposide-reduced myosin light chain kinase expression, suggesting that geniposide ameliorated barrier dysfunction via AMPK-mediated inhibition of the MLCK pathway [86]. Activation of glucagon-like peptide-1 (GLP-1) receptor exerts a range of cardioprotective effects. Geniposide is an agonist of the GLP-1 receptor. Activation of AMPKα and inhibition of the mammalian target of rapamycin, ERK and endoplasmic reticulum stress were observed in hypertrophic hearts treated with geniposide [57].

### 3.4 Apoptosis regulation

Genipin induced hepatoma cell apoptosis, which was mediated by ROS/c-Jun NH2-terminal kinase-dependent activation of the mitochondrial pathway [87]. In the anti-tumor mechanism, the activation of JNK may result in an increase in p53 protein level and lead to the mass accumulation of bax protein. Genipin-induced apoptosis was associated with activation of the c-Jun NH2-terminal kinase and p53 protein in HeLa cells [88]. Geniposide protected rat insulinoma cells from apoptosis in high-glucose concentrations, and these effects were associated with an increased apoptosis-related Bcl-2/BAX protein ratio [89]. Geniposide effectively induced adjuvant-induced arthritis fibroblast-like synoviocyte apoptosis by regulating apoptosis-related gene expression, as shown by a decreased Bcl-2 mRNA level and increased Bax and caspase 3 mRNA levels [90]. β-Cell apoptosis is considered to be a major cause of β cell loss in diabetes. Geniposide prevented oxidative stress-induced neuron apoptosis, and improved glucose stimulated insulin secretion by activating glucagon-likepeptide1 receptor (GLP-1R) in INS-1 cells [91]. Penta-acetyl geniposide transduced the apoptotic signals through PKCδ activation and the downstream cascades of JNK/Jun phosphorylation, FasL/Fas elevation, and the subsequent activation of caspase 8 and caspase 3 [92, 93].

### 3.5 Regulation of glucose metabolism and lipid metabolism

In diet-induced hyperlipidemic rats, a 10-day treatment with crocin significantly reduced serum triglycerides, total cholesterol, low density lipoprotein cholesterol and very low density lipoprotein cholesterol levels following a daily dose range of 25-100 mg/kg, which demonstrated that crocin exerted its hypolipidemic effect by inhibiting pancreatic lipase, leading to malabsorption of fat and cholesterol [22]. In streptozotocin-induced diabetic rats, geniposide increased insulin serum level, decreased glucose level and affected the levels of total cholesterol and triglycerides [94]. Moreover, a study showed that GF improved insulin secretion and lowered plasma lipids in steroid-induced insulin resistant rats. Intracellular signaling proteins including peroxisome proliferator-activated receptor and insulin receptor substrate-1 were elevated by GF [96, 96]. It was shown that geniposide might be a potential drug for hypertriglycemia in diabetes. In addition, genipin, has been shown to alleviate age-related insulin resistance, hyperinsulinemia, hyperglyceridemia and hepatic oxidative stress in aging rats [73]. Research on geniposide’s regulation of hepatic glucose production in HepG2 cells showed that geniposide inhibited hepatic glucose production in a dose-dependent manner. Different concentrations of geniposide stimulated AMPK, forkhead box class O1 phosphorylation and acetyl coenzyme A synthetase. In addition, the enzyme activities of glucose-6-phosphatase and phosphoenolpyruvate carboxykinase were suppressed by geniposide [97]. It has also been reported that the regulation of geniposide on diabetic cell adhesion and vascular injury might be related to its anti-oxidative activity and NF-κB signal pathway activation [98]. A clinical study showed that Gardenia intake with exercise had a positive effect on body composition and energy metabolism regulating hormones, characterized by the decrease of the visceral fat area in middle-aged obese women [99]. In estrogen-deficient rats, GF combined with Artemisia princeps Pamp and Leonurus japonicas Houtt exhibited increased hepatic peroxisome proliferator-activated receptor-γ coactivator-1α expression, which increased the number of genes involved in fatty acid oxidation and decreased fatty acid synthesis. These results suggested that GF may have potential as a therapeutic agent in treating postmenopausal symptoms [100].

## 4. Conclusions and Future Studies

In this review, we summarized the therapeutic potential and effective components of the Chinese herb Gardeniae fructus in the treatment of age-related diseases. The anti-aging properties of GF and its effective components are associated with inflammation, mitochondrial dysfunction and oxidative stress. Although the mechanisms of GF and its bioactive anti-aging components have been explored, there have been few studies examining the effects of GF and its bioactive components on central nervous system diseases. In addition, the main components of GF have a dose-dependent effect on anti-aging, but the potential side-effects of these components have not yet been fully studied. In addition to the longevity effects, many studies on GF and its effective components have been conducted to demonstrate their other health promoting properties, such as anti-cancer activity based on its anti-senescence effect. To the best of our knowledge, several experiments have shown the neuroprotective activities of GF *in vitro* but few studies have examined its effects *in vivo*. Over the last decade, there have been many studies investigating the therapeutic potential of GF and the use of its constituents as natural supplements for combating the aging process. Findings from these studies could lead to the development of GF preparations as new therapeutic agents for the treatment of age-related diseases.
